# Analysis of awareness of health knowledge among rural residents in Western China

**DOI:** 10.1186/s12889-015-1393-2

**Published:** 2015-01-31

**Authors:** Fang Yuan, Dongfu Qian, Chenglong Huang, Miaomiao Tian, Yuanxi Xiang, Zhifei He, Zhanchun Feng

**Affiliations:** School of Medicine and Health Management, Tongji Medical College, HuaZhong University of Science and Technology, Wuhan, HuBei Province China; College of Medical Administration, Nanjing Medical University, Nanjing, Jiangsu Province China; College of Laboratory Medicine, Chongqing Medical University, Chongqing, China; Institute of Medical Information, Center for Health Policy and Management, Chinese Academy of Medical Sciences, Beijing, China

**Keywords:** Health education, Awareness of health knowledge, Education, Rural areas, Western China

## Abstract

**Background:**

Lifestyle diseases could be prevented and controlled by disseminating health knowledge. This study explored the health knowledge awareness and the impact factors of health knowledge awareness, and the way people received health knowledge in western China.

**Methods:**

We undertook a cross-sectional survey in 8 counties, 24 townships and 72 villages from July 2011 to April 2012 in Inner Mongolia, Xinjiang, Chongqing and Qinghai in China. Collected data, which were publicly available, consisted of two parts, namely, socio-demographic information and the 1466 corresponding rural residents’ awareness and the approach of health knowledge. Analysis of Variance (ANOVA) was used to explore the impact factors of health knowledge awareness. Multiple linear regressions was then applied to examine the potential predictors of health knowledge awareness.

**Results:**

Four predictors-age (negative factor), educational level (positive factor), distance from home to the nearest medical institution (negative factor) and annul disposable household income (negative factor) were in the final liner regression model (p < 0.05). The results showed that awareness of health knowledge associated with risk factors was the highest (58.85%). The highest awareness rate of health knowledge is the title “Whether secondhand smoke is harmful to myself” (69.78%) and the lowest title is “Whether eating with hepatitis B patients will be infected Hepatitis B” (21.69%). The main way to receive health knowledge was traditional way such as doctors (80.45%). About more than half of the residents received health knowledge through television, video, newspaper and magazines (65.78%), family members, neighbors (67.38%) and the village health bulletin boards (53.16%).

**Conclusion:**

Health knowledge awareness of rural residents was quite low and the way of receiving health knowledge was simple and traditional. One of the critical factors was education level. Direct results showed that lower income families always obtained higher health knowledge level than the rich families. The main way to receive health knowledge was traditional ways. In the process of health education, different means of education should be adopted for different groups so as to achieve ideal effect. Potential interventions may be different from education process which should be adapted to different income level families.

**Electronic supplementary material:**

The online version of this article (doi:10.1186/s12889-015-1393-2) contains supplementary material, which is available to authorized users.

## Background

A large number of medical researches confirmed that 60% of the diseases were caused of unhealthy lifestyles [1]. In fact, unhealthy lifestyle related diseases can be prevented and controlled. Changing from disease treatment to disease prevention and health management is the trend within the global health progress; especially health communication and education are being paid more attentions [2].

Health promotion is based on health education, and health knowledge is the foundation of health education [3]. At present, the proportion of residents' health literacy is less than 7% in China [4]. Improving the health literacy of citizens is helpful for improving reasonable treatment ability of residents, promoting the rational use of existing medical and health resources, improving consciousness of residents in prevention and self-health care, making the right judgments for residents to their own health and dealing with public health emergencies scientifically [5].

Research results from D and abroad showed that social class, as the most decisive factor, affects health and life expectancy [6]. Therefore, there is a large gap between different social classes on health fairness and health services utilization. Previous research had shown that the influencing factors of health were not only the low income but also the unfairness of income distribution [7]. The study also found that low socioeconomic status was closely related to the acceptance of educational level [8,9]. Adults, who lack of formal education, would increase the probability of unemployment, which will be tight in terms of economy, and as a result it will lead to poor health [10].

The major influencing factors about the spread of health knowledge are the ways people receiving health knowledge. There are some reasons. Firstly, the higher media acceptance arrival rate and repetition rate, the greater possibility of accessing to information they had [11]. Secondly, authoritativeness of source is important. The more reliable and authoritative the source is, the more persuasive information is, and then the greater possibility to get attitude changed [12]. The third one is the transmission efficacy of medium. The more appeal of media dissemination and the stronger affinity, the higher transmission efficiency, the more conducive to the change in attitudes [13].

The rural residents in the west part of China live mainly in the highlands, and their life environment are affected by low pressure hypoxia. Firstly, because of the environmental and regional impacts, basically they do not have sufficient vegetables and fruits in all seasons, and diets are mainly composed of meat and milk. Therefore, the diet contains excess salt and high energy, but lacks multi-vitamins, which increases the potential risk of body nutrients imbalance, hyperlipidemia, and high blood pressure. Secondly, due to the cold living environment, rural residents in the northwest minority areas smoke heavily and consume alcohol excessively [14]. Thirdly, the residents live in rural areas have lower economic and education levels, and more limitation in understanding of health knowledge. Finally, medical supplies can not meet the demand of the masses, and drugs are scarce in health organization of counties and villages. Health technician resource is insufficient, meanwhile, the service radius is big and prevention task is heavy [15]. Thus, the spread of health knowledge plays an important role for health of rural ethnic minorities in northwest China.

Currently, many studies within health knowledge and health education have been presented, however, these studies only focused on a specific range of knowledge about the situation. Studies about comprehensive health knowledge of the entire population are limited. This study aims to research the health knowledge awareness of rural residents and health knowledge that the residents are currently using in western China. The relationship among reasonable evaluation of health demand, improving the health promotion activities and health level of residents are present simultaneously.

## Method

### Study population

**Figure 1:**
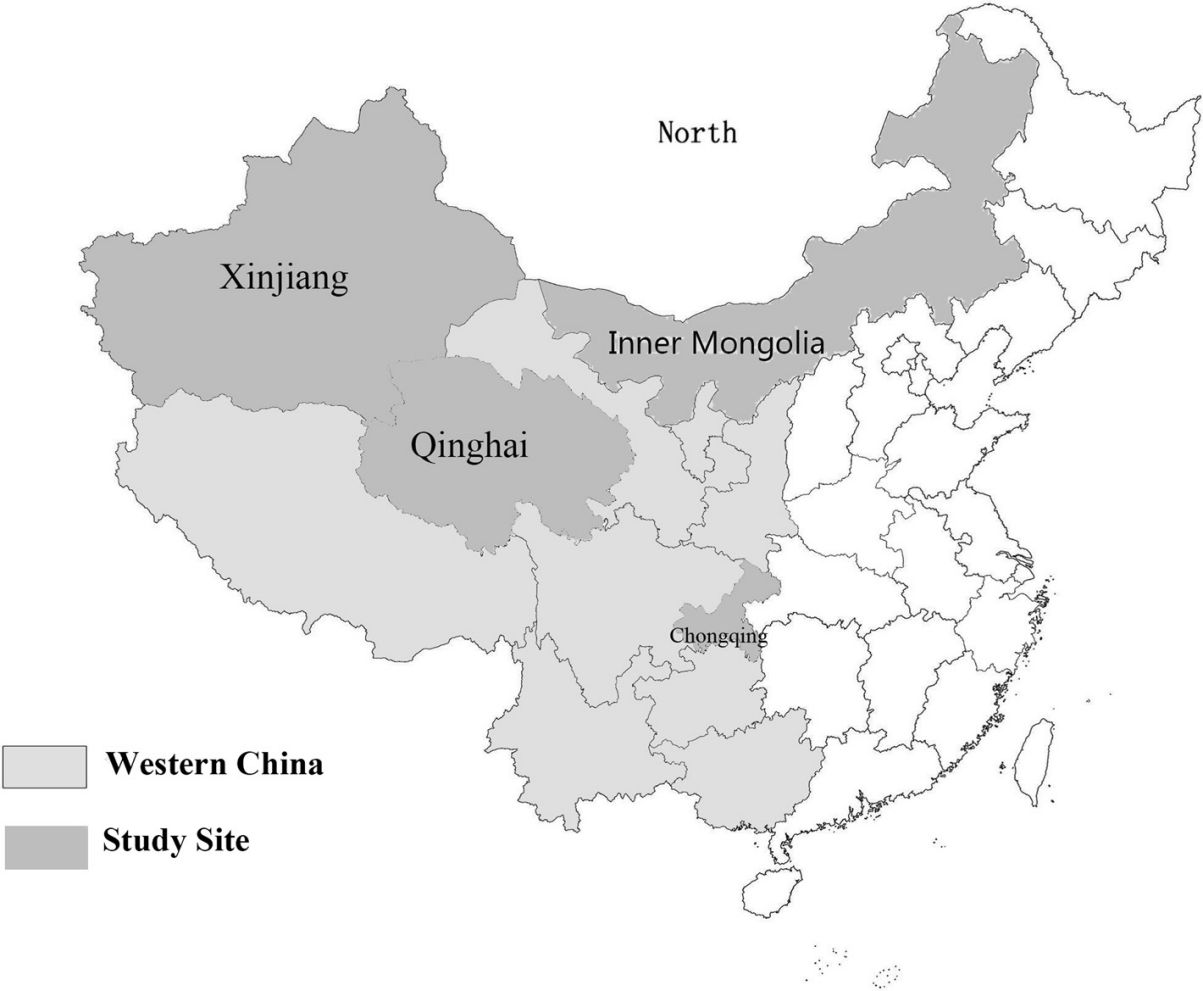
**Distribution map of study sites.**

The data were collected from a cross-sectional survey which was conducted from July 2011 to April 2012. The sampling procedure involved five levels: province/municipality, county, township, village, and respondent. Firstly, all the 12 provinces/municipalities in western China were divided into four groups according to Chinese statistics yearbook 2012, and one province/municipality was selected at random from each group. Inner Mongolia (in first group), Chongqing (in second group), Xinjiang (in third group) and Qinghai (in last group) were chosen as the study sites (Figure 1). Secondly, two counties were randomly selected from each of the four selected study sites’ counties as the sample counties (4×2 = 8 counties selected). Thirdly, the townships of the sample countries were divided into three categories based on their distances from the county town (4×2×3 = 24 township selected). Fourthly, three villages per sample townships were randomly chosen from the category as the sample village (4×2x3×3 = 72), for a total of 72 villages. Fifthly, 10 to20 rural residents were selected randomly from each village. A total of 1466 rural residents was investigated and completed the survey. The overall refusal rate was 0. No one refused to our investigation. The study was randomized at municipality level and the proportion of education level and household income were consistent from statistical yearbook of the sample regions [16]. Due to the large number of outside worker, the proportion of sexual,age,occupation had the difference between statistical yearbook,however some domestic research sampling result is consistent with our study [17-19].

### Quality control

Face-to-face interviews were conducted by interviewers with each participant, and the study purpose was clearly explained to them. Students from the School of Medicine and Health Management of Tongji Medical College and staff of local health care institutions were recruited and trained as interviewers. Items and response choices were read to participants who had difficulty in reading (because of illiteracy or poor vision). All participants signed a written informed consent in this study.

The research staff training: the research staff had a full-time and on-the-job training to guarantee they were acquainted with the target, significance and measures of the investigation.

The arrangement of the data: all data were entered in duplicate into the EpiData Info version 3.1 databases and statistics program (Atlanta, Georgia, USA). Data entry screens were used to revise incorrect entries. Statistical analysis was conducted using PASW statistics 18.0 (SPSS, Chicago, IL, USA).

This study was approved by the Ethics Committee of Tongji Medical College, Huazhong University of Science and Technology (IORG No: IORG0003571) and informed consent was obtained from all participants in the study.

### Questionnaire

The questionnaire was distributed in simplified Chinese and contained a combination of open- and closed-ended questions. The questionnaire consisted of two parts, socio-demographic information and health knowledge awareness (Additional file 1).

Socio-demographic information included sex, age, educational level, employment status, distance from home to nearest medical institutions, and family economic conditions. The health knowledge awareness involved health knowledge level of residents, the factors that influenced health knowledge, and approach of achieving health knowledge. In this study, health knowledge items were developed by referring to the 2008 Chinese Citizens’ Health Literacy Survey [20] and health brochures delivered to residents in rural China. On the basis of these materials, we selected nine life behavioral risk factors which were closely linked to everyday life and had a higher awareness as the health knowledge problem. The main indicators of dividing health knowledge included risk factors, prevention knowledge and understanding of health. The total number of correct answers was calculated as the overall health knowledge score. No points would be awarded for incorrect or missing answers or the answer “don’t know.” The approaches of achieving health knowledge included five ways: radio, television, newspapers, magazines and other media, SMS messages, Internet and other media to obtain, doctors, health promotion materials, village health bulletin boards, health care seminars and other channels to be acquired and via family members, neighbors or friends to understand health care knowledge.

### Statistical methods

Socio-demographic information on residents surveyed was summarized using descriptive statistics. Health knowledge level of residents, health knowledge acquisition and approach of achieving health knowledge were summarized using descriptive statistics. Analysis of Variance (ANOVA) was used to explore the impact factors (sex, age, educational level, occupation, and distance from home to nearest medical institutions and family economic conditions) of health knowledge awareness (p-value < 0.05). Multiple linear regression was then applied to examine the potential predictors of health knowledge awareness. The dependent variable was the score of health knowledge. Independent variables included: age, education level, and distance from home to the nearest medical institutions and annul disposable household income.

## Results

### Socio-demographic characteristics of participants

The socio-geographic data of the study regions in 2011: the population of rural Inner Mongolia was 10.77 million; the population density of it was 5.43‰. The Rural per capita disposable income was 6641.6 yuan in rural Inner Mongolia. The population of rural Xinjiang province was 12.04 million; the population density of it was 4.42‰. The Rural per capita disposable income was 5442 yuan in Xinjiang province. The population of rural Qinghai was 3.06 million; the population density of it was 6.12‰. The Rural per capita disposable income was 4608.47 yuan in rural Qinghai province. The population of rural Chongqing was 13.13 million; the population density of it was 6.71‰. The Rural per capita disposable income was 6480.4 yuan in rural Chongqing province [16].

Among 1466 survey respondents, the number of females was 883 (60.2%), males 583 (39.8%). Age distribution: 66 and over group had the largest proportion (33.4%). The average of age was 55.72, the range of age was 13to 93. Educational level: less than 6 years of elementary education group had the largest proportion (40.7%) and the smallest proportion was college group (1.1%). Employment status: more than two-thirds (74.9%) were farmers. 85.5% of the residents lived less than 1 km near to medical institutions. More than two-third families economic situation of participants (69.8%) was low (Annual household income < CNY 22000). The average of annual household income was 19125.76 yuan, and the range of it was 500 yuan to 150000 yuan. The socio-demographic characteristics of participants indicated that residents surveyed were less educated with low household income (Table 1).

**Table 1:** **Socio-demographic characteristics of rural residents investigated in Western China**

**Characteristics**	**Participants (n = 1466)**	**Percent (%)**
**Gender**		
Male	583	39.8
Female	883	60.2
**Age**		
25 and blow	69	4.7
26 to35	178	12.1
36 to 45	156	10.6
46 to 55	226	15.4
56 to 65	347	23.7
66 and over	490	33.4
**Education level**		
less than 6 years of elementary education	597	40.7
Elementary	452	30.8
Middle school	329	22.4
high school and above	72	4.9
College	16	1.1
**Occupation**		
Farm	1098	74.9
Migrant worker^a^	56	3.8
Self-employed^b^	55	3.8
Factory workers	53	3.6
Retirement	74	5.0
Others	130	8.9
**Distance from home to nearest medical institutions**		
Less than 1 km or equal to 1 km	1253	85.5
Further than	213	14.5
**Annul disposable household income group** ^**c**^		
Less than CNY 22000	1017	69.8
CNY 22001 to CNY 29000	132	9.0
CNY 29001 to CNY 37000	136	9.0
CNY 37001 to CNY 48000	61	4.1
CNY 48001 and above	120	8.1

Note: ^a^migrant worker indicates rural citizens who leave their villages and go into the cities to do off-farm work.

^b^self-employed laborers refer to people who run a private, small-scale business, such as vendors, food-shop owners, repair-shop owners, and rice-noodle sellers.

^C^reference to 2012 China statistical yearbook.

### The awareness of health knowledge

Majority residents (88.34%) did not know the route of transmission of hepatitis B. Almost 83.67% of residents still thought that physical health was the concept of health. However, the awareness of overall health knowledge level was still relatively poor (The average correct rate: 46.47%) (Table 2).

**Table 2:** **Health knowledge level of rural residents in westen China**

**Question**	**Indicator**	**The number of right (n = 1466)**	**Percent (%)**
Whether secondhand smoke is harmful to myself	Risk factors	1023	69.78
Whether salty food will cause high blood pressure	Risk factors	916	62.48
Whether obese people are more susceptible to diabetes	Risk factors	571	38.95
Whether excessive drinking will harm the function of the liver	Risk factors	941	64.19
Whether eating with hepatitis B patients will be infected Hepatitis B	Prevention knowledge	318	21.69
Whether eating the fruit and vegetables which were picked freshly in the ground and brushed by hand	Prevention knowledge	426	29.06
Whether vaccination for children in order to prevent infectious diseases	Prevention knowledge	989	67.46
Whether anemia is related with Iron deficiency	Prevention knowledge	561	38.27
Whether health is neither fat nor thin, eat well, sleep well and not sick	Understanding of health	386	26.33
The average correct rate	-		46.47

To estimate the combined effect of a single factor and control confounding factors effectively, single-factor analysis was used to explore the influencing factors of health knowledge score stratification. Dependent variable was health knowledge score and independent variables were gender, age group, educational level, occupation, distance from home to the nearest medical institutions, and family economic conditions. Each question score were assigned 1 point, add up to a total of 9 points. Residents’ health knowledge level was considered as the population average score. Analysis indicated the awareness of health knowledge was related to age (p = 0.000 < 0.05), education (p = 0.000 < 0.05), distance from home to nearest medical institutions (p = 0.010 < 0.05) and Annual household income (p = 0.002 < 0.05). It shows that knowledge levels declined steadily with increasing age, further distance from home to the nearest medical institution and the more disposable household income. But the knowledge level increased with higher educational attainment (Table 3).

**Table 3:** **Influencing factors of Health knowledge level of rural residents in Western China**

**Factors**		**Population average score**	**F**	**P**
Gender	Male	4.67	0.145	0.703
	Female	4.62		
Age	25 and blow	6.13	43.583	0.000
	26 to35	5.72		
	36 to 45	5.41		
	46 to 55	4.91		
	56 to 65	4.60		
	66 and over	3.70		
Educational level	less than 6 years of elementary education	4.12	16.522	0.000
	Elementary	4.92		
	Middle school	4.99		
	high school	5.17		
	College and above	5.58		
Employment status	Farm	4.57	1.937	0.085
	Migrant worker^a^	4.95		
	Self-employed^b^	4.87		
	Factory workers	5.23		
	Retirement	5.05		
	Others	4.54		
**Distance from home to nearest medical institutions**	Less than 1 km or equal to 1 km	4.70	6.071	0.014
	Further than	4.30		
**Annul disposable household income group** ^**c**^	Less than CNY 22000	5.35	120.590	0.000
	CNY 22001 to CNY 29000	3.42		
	CNY 29001 to CNY 37000	3.20		
	CNY 37001 to CNY 48000	2.92		
	CNY 48001 and above	2.48		

Note: ^a^migrant worker indicates rural citizens who leave their villages and go into the cities to do off-farm work.

^b^self-employed laborers refer to people who run a private, small-scale business , such as vendors, food-shop owners, repair-shop owners, and rice-noodle sellers.

^C^reference to 2012 China statistical yearbook.

### Predictors of health knowledge awareness among rural western Chinese residents

Because the bivariate effects of the predictors on the dependent variable were probably confounded by other factors, multiple liner regression analysis was further used to examine the predicting effect of each potential predictor identified in the bivariate analysis to adjust for the effects of other confounding variables. Four predictors- age, educational level, distance from home to nearest medical institution and annul disposable household income- were retained in the final liner regression model to predict health knowledge awareness among rural residents in western China (Table 4). The whole model test results had significant (F = 103.876, p = 0.000 < 0.05) and the models had qualified fitting degree and applicability (R^2^ = 0.221). All variables which were significant in bivariate analysis were entered for multivariate analysis and all they were significant (p < 0.05). Among all the significant predictors, distance from home to nearest medical institution had a highest contribution in the model (b = −0.510) and it was a protect contribution in the model. Age and annul disposable household income were both protect contribution (b = −0.041 and −0.418) the educational level was the only danger contribution in the model (b = 0.251).

**Table 4:** Result of multivariate liner regression analyses*examining factors associated with alcohol use among rural Chinese residents

**Model**	**Unstandardized C**		**t**	**95% C.I.**		**P-value**
	**B**	**Std.Error**		**Lower**	**Upper**	
Age	−0.041	0.003	−13.098	−0.047	−0.035	0.000
Educational level	0.251	0.054	4.651	0.145	0.357	0.000
Distance from home to nearest medical institutions	−0.510	0.144	−3.548	−0.792	−0.228	0.000
Annul disposable household income group^c^	−0.418	0.037	−11.211	−0.491	−0.345	0.000

*The input variable: age, educational level, distance from home to nearest medical institutions, annul disposable household income group.

### The approach of receiving health knowledge

Analysis indicated that most people received health knowledge from doctors, and more than half of residents (65.78%) got health knowledge through radio, television, newspapers and magazines. Only 21.67% of residents received health knowledge from SMS or Internet. It indicated that it was widely hoped to get health knowledge through traditional ways such as doctors (Table 5).

**Table 5:** **The approach of achieving health knowledge of rural residents in western China**

**The approach of achieving health knowledge**	**Participants (n =1466)**	**Percent (%)**
Through radio, television, newspapers, magazines	964	65.78
Through SMS, Internet	318	21.67
Through doctors	1179	80.45
Through health promotion materials, village health bulletin boards, health care seminars	779	53.16
Through family members, neighbors or friends	988	67.38

## Discussion

This study compared different demographic characteristic, distance from home to the nearest medical institute with health knowledge awareness, and different ways that people receive health knowledge. In this study, we have not found any evidence for the impact of occupation and gender. The probability of high health knowledge was younger age, high educational level, nearer distance from home to nearest medical institution and low annul disposable household income of rural residents in western China. As we found previously, high health knowledge level people were more likely to be high education level [21,22]. In our study, we had the same results, such as less than 6 years of elementary education group got the lowest score (4.12) and higher education level had the higher score (education level: b = 0.251,p = 0.000). This could be due to the higher level of literacy, receptivity and comprehensive ability [23,24].

The health problem of the elderly is at the world’s attention, especially the elderly health education issues [25]. According to the data analysis, rural residents aged over 65 had the lowest level of health knowledge awareness (score = 3.70, p = 0.000 < 0.05). Due to the lack of ability and knowledge to the elderly with their own life behavior change, they become the type of groups t with most difficult to control in their own health and carry out in healthy education in rural areas [26,27]. Even though health care for 65 - year – old and above has been listed as a essential public health service, it is not obvious of implementing improvement effect in the progress and rural elderly residents’ health problems to be solved [28].

Previous researches found that the family economic had a positive effect to health knowledge concerning that high economic condition family would pay more attention and spend fees to education and health [9,10,26,27]. However, we found that annul disposable household income had a negative correction with health knowledge awareness which is different from the previous studies. The survey found that high degree of health knowledge level lower than that of low-income earners in western rural area (Annual household income ≥ CNY 48001, score = 2.48; annual household income < 22000, score = 5.35. p = 0.000 < 0.05, b = −0.418). Even though the infector had a little influence on the liner regression model, it is significant. There are three possible reasons. First, in China, high earners’ malignant tumor detection rate is 73 times higher than the overall level of the incidence of malignant tumors, the average detection rate has more than 60% of dyslipidemia and cardiovascular death rate is 1 time more than China’s total population [29]. Previous studies showed that high earners carbohydrate intake was lower (67.6% of RDA) than RDA (The Chinese nutrition society recommended dietary supply), fat and protein intake were significantly higher (141% and 160.3% of RDA) than RDA [30]. Although the quality of life and consumption of high-income people were higher than that of low-income people, the high-income people were more likely to ignore their health and paid less attention to their health risk behaviors [31]. Higher income to become rich way is often go out to work or engage in labor intensive processing industry in the country and In the case of abundant material life and not timely enrich the spiritual life [32]. The level of education has not been improved. Second, due to the accelerating rhythm of life for not paying attention to a person’s own health, but also did not get the guidance of professional about health care in a timely manner, obviously their thoughts still generally stay to think smoke and drink is for the sake of pleasure, buried a serious hidden danger to their health [33]. Third, China has implemented a twelve-year compulsory education since 2006. All students before the university education are free. In rural China, people think that knowledge can change destiny. The poor families would borrow money to go to college. So that family economy had no significant correlation with education level.

Due to the low education level of rural residents, health knowledge received channel is relatively single and traditional [31], the main health knowledge received channels via traditional ways such as doctors (80.45%) and radio, television, newspaper, magazines (65.78%). The modern channel such as SMS and Internet had the lowest utilization (21.76%). This result coincides with that the closer distance from home to nearest medical institution as the higher score of health knowledge. Due to most residents received health knowledge from doctors, so that medical institutions were closer to home, the more convenient they went there, and the higher frequency of communication they had. In addition, the dispersion of rural residents living in western rural area is relatively less people contact with each other, so that they receive insufficient health knowledge [34]. Therefore to get health knowledge mainly relied on doctors and television radio newspapers and other common media. However, the examination of the health knowledge of drug content of television, broadcast, newspapers and so on is not strict, leading to a lot of rural residents is deceived, and they never trust the health coverage on TV. In the village clinics and township hospitals and other places set up a disease prevention publicity column and brochures and other health education activities used as the way of application requirements and actual use are relatively low. Health bulletin boards and handbook are very popular in rural China. They are set up and managed by village committees and the content includes election dates, natural disaster warnings, and physical examination times. However, it plays a minor role for the improvement of health promotion especially drinking behavior. There may be two reasons: first, few aspects of the content related to the knowledge which the residents need; second, rural resident educational level is too low to get health knowledge through the bulletin board effectively.

One of the limitations to this study is that some predictors found by other researches (e.g. whether have chronic diseases) were not included. Another research limitation is that we have not investigated residents’ physical condition for example, blood pressure, height, weight, or body mass index, and the data of past history or family history of participants. Another research limitation is the use of closed-ended questions in the health knowledge test, which may have allowed participants to guess the correct answer. In addition, the results of this study can have further verification in the future research by increasing the sample size.

## Conclusion

In summary, health knowledge awareness of rural residents is quite low and the receiving way of health knowledge is simple and traditional. One of the critical factors was education level, and knowledge level increased with higher education level. Direct results showed that lower income families always obtained higher health knowledge level than the rich families. And the residents who lived within 1 km to the nearest medical institutions have higher awareness of health knowledge than those who lived further than 1 km to the nearest medical institutions. Besides, health knowledge level declined steadily with increasing age. The main way to receive health knowledge was traditional ways such as doctors. Potential interventions may be different from education process which should be adapted to different income level families. For residents who live far from the medical institutes, village doctors could send some text about health knowledge to them regularly. For those whose educational level is not high, the doctors could use more comprehensible way to teach health knowledge to them. In the process of health education, different means of education should be adopted for different groups so as to achieve ideal effect.

## Additional file

Additional file 1:
**Questionnaire for residents in rural western China.**

## References

[1] Physical activity and healthy eating the global strategy (2004). World Health Organization.

[2] Jianfeng B (2012). On Health Communication and Public Health Behavior Optimization in China. PhD thesis.

[3] Chaponniere PA, Cherup SM, Lodge L (2013). Measuring the Impact of Health Education Modules in Cameroon, West Africa. J Transcult Nurs.

[4] China Health and family planning committee (2009). Chinese residents’ health literacy survey report for the first time.

[5] Sabir SA, Hassan F, Zain-ul-Abideen (2013). Improving health literacy in Pakistan--“a new oil in old lanterns”. J Pak Med Assoc.

[6] Chan MF, Taylor BJ (2013). Impact of demographic change, socioeconomics, and health care resources on life expectancy in Cambodia, Laos, and Myanmar. Public Health Nurs.

[7] Bosma H, Gerritsma A, Klabbers G, van den Akker M (2012). Perceived unfairness and socioeconomic inequalities in functional decline: the Dutch SMILE prospective cohort study. BMC Public Health.

[8] Petersen PE, Kwan S (2011). Equity, social determinants and public health programmes–the case of oral health. Community Dent Oral Epidemiol.

[9] Bohnert A, Burdette K, Dugas L, Travers L, Randall E, Richards M (2013). Multimethod analyses of discretionary time use and health behaviors among urban low-income african-american adolescents: a pilot study. J Dev Behav Pediatr.

[10] Lui CK, Chung PJ, Wallace SP, Aneshensel CS (2013). Social Status Attainment During the Transition to Adulthood. J Youth Adolesc.

[11] Anikeeva O, Bywood P (2013). Social media in primary health care: Opportunities to enhance education, communication and collaboration among professionals in rural and remote locations. Aust J Rural Health.

[12] Cole A (2013). Public has fewer worries about sharing health information than other personal data. BMJ.

[13] Galanis P, Sourtzi P, Bellali T, Theodorou M, Karamitri I, Siskou O, Charalambous G, Kaitelidou D (2013). Public health services knowledge and utilization among immigrants in Greece: a cross-sectional study. BMC Health Serv Res.

[14] ZHAO O. Qinghai yushu Tibetan residents living habits of the influencing factors of health. Journal of qinghai normal university (natural science edition). 2001(02):50-53.

[15] Deying L, Haiyun Y. The western rural areas the main problems of the basic medical and health resources allocation and the countermeasure analysis. Western Econ Manage. 2011(04):28-30+62

[16] National Bureau of Statistics of the People’s Republic of China (2011). 2011 China Statistical Yearbook.

[17] Chen C. The main factors which restrict the development of the western rural population analysis. Sichuan Agric Sci Technol. 2014(07):8-10

[18] Xu C. The western rural left-behind women family stress and its influencing factors is analyzed. Popul Econ. 2010(01):73-78

[19] Yang X. Analysis of Remained Elderly in the Western Rural Areas. Value Eng. 2011(13):12-14.

[20] National Health and Family Planning Commission of the People’s Republic of China Notice from the Ministry of Health: basic knowledge and skills in health literacy of Chinese residents, Volume 2012: 1-3.

[21] Kobel S, Wirt T, Schreiber A, Kesztyus D, Kettner S, Erkelenz N (2014). Intervention effects of a school-based health promotion programme on obesity related behavioural outcomes. J Obes.

[22] Wang W, Hou Y, Hu N, Zhang D, Tao J, Man Y (2014). A cross-sectional study on health-related knowledge and its predictors among Chinese vocational college students. BMJ Open.

[23] Duma OO, Rosu ST, Manole M, Petrariu FD, Constantin B (2014). Disparities in the access to primary healthcare in rural areas from the county of Iasi - Romania. Rev Med Chir Soc Med Nat Iasi.

[24] Lim W, Chuang DF, Chue KM, Lee DZ, Leong NJ, Ng ZG (2014). Stroke literacy in singapore: data from a survey of public housing estate residents. Ann Acad Med Singapore.

[25] Hanlon JT, Semla TP, Schmader KE (2014). Medication misadventures in older adults: literature from 2013. J Am Geriatr Soc.

[26] Boyle PA, Yu L, Wilson RS, Segawa E, Buchman AS, Bennett DA (2013). Cognitive decline impairs financial and health literacy among community-based older persons without dementia. Psychol Aging.

[27] Lu X, Wang L, Chen H. Health education in community elderly health promoting new mode analysis of the difficulties and countermeasures. J Nurs Res. 2011(36):123-24.

[28] CPC Central Committee and State Council on Deepening the views of medical and health system. Beijing: Chinas State Council; 2009.

[29] Human resource management, High income = high rates? Body is the capital of the revolution. J Hum Resour Manag. 2013(03):158.

[30] Cuifeng Z, Suhuan B, Yan L, Xiangong Z, Hongyan L, Hengjiao C, Wei H. Shenzhen high earners relationship between diet and metabolic syndrome. Chin J Public Health. 2007(08):949-51.

[31] Satariano WA, Kealey M, Hubbard A, Kurtovich E, Ivey SL, Bayles CM, et al. Mobility disability in older adults: at the intersection of people and places. Gerontologist. 2014;00(00):1–11. 10.1093/geront/gnu094 PMC4873765 25326342

[32] Enrun C,Yingfang G. Basic strategy for the sustainable development of agricultural economy. Agric Econ. 2012(09):21-22.

[33] Anping C. High income will be more healthy? The new evidence from China. Econ Finance Trade. 2011(01):26-33.

[34] Qiuzhuang W. The status of rural health education present situation and practical countermeasures. J Pract Med Tech. 2007;14(12):1643–4.

