# Supplementary material for: Analysis of awareness of health knowledge among rural residents in Western China
Source: BMC Public Health. 2015 Jan 31;15:55. doi: 10.1186/s12889-015-1393-2 (PMC4320617; doi:10.1186/s12889-015-1393-2)
Supplement: Additional file 1: — Questionnaire for residents in rural western China. [file 12889_2015_1393_MOESM1_ESM.doc]

# Additional files

### Additional file 1 –Questionnaire for residents in rural western China

| **Part A Basic personal information** | | **Answer** |
| --- | --- | --- |
| 1 | Date：______________ |  |
| 2 | Whether the questions are answered by yourself ?  (1) Answered by myself (2) Answered by others |  |
| 3 | Your gender：(1)Male (2)Female |  |
| 4 | Your age(year) : ______________ |  |
| 5 | Education level：(1) less than 6 years elementary study (2)Elementary  (3) Middle school　(4) high school (5)college and above |  |
| 6 | Occupation：(1)farmer (2)migrant worker (3)self-employed (4)Factory workers (5)Retirement (6)others |  |
| 7 | How far is your home from nearest medical institutions: (1) less than 1km  (2)1km~2km (3)2km~3km (4)3km~4km (5)4km~5km (6)further than 5km |  |
| 8 | How much is your annul disposable household income: ___________(yuan) |  |
| **Part B Health knowledge** | |  |
| 1 | **Risk factors** |  |
| 1.1 | Whether secondhand smoke is harmful to myself  (1) true; (2)false; (3) I don’t know |  |
| 1.2 | Whether salty food will cause high blood pressure  (1) true; (2)false; (3) I don’t know |  |
| 1.3 | Whether obese people are more susceptible to diabetes  (1) true; (2)false; (3) I don’t know |  |
| 1.4 | Whether excessive drinking will harm the function of the liver  (1) true; (2)false; (3) I don’t know |  |
| 2 | **Prevention knowledge** |  |
| 2.1 | Whether eating with hepatitis B patients will be infected Hepatitis B  (1) true; (2)false; (3) I don’t know |  |
| 2.2 | Whether eating the fruit and vegetables which were picked freshly in the ground and brushed by hand  (1) true; (2)false; (3) I don’t know |  |
| 2.3 | Whether vaccination for children in order to prevent infectious diseases  (1) true; (2)false; (3) I don’t know |  |
| 2.4 | Whether anemia is related with Iron deficiency  (1) true; (2)false; (3) I don’t know |  |
| 3 | **Understanding of health** |  |
| 3.1 | Whether health is neither fat nor thin, eat well, sleep well and not sick  (1) true; (2)false; (3) I don’t know |  |
| Part C The ways of receiving health knowledge | |  |
| 1 | Do you receiving knowledge through radio, television, newspapers, magazines usually? (1)Yes (2)No |  |
| 2 | Do you receiving knowledge through SMS, Internet usually?  (1)Yes (2)No |  |
| 3 | Do you receiving knowledge through doctors usually?  (1)Yes (2)No |  |
| 4 | Do you receiving knowledge through health promotion materials, village health bulletin boards, health care seminars usually?  (1)Yes (2)No |  |
| 5 | Do you receiving knowledge through family members, neighbors or friends usually? (1)Yes (2)No |  |
